# Supplementary material for: Content-rich biological network constructed by mining PubMed abstracts
Source: BMC Bioinformatics. 2004 Oct 8;5:147. doi: 10.1186/1471-2105-5-147 (PMC528731; doi:10.1186/1471-2105-5-147)
Supplement: Additional File 2 — The original results of the above study (non-essential files are deleted to keep the file size under the limit set by BMC bioinformatics). [file 1471-2105-5-147-S2.bz2 › chilibotAdditionalFile2/dip05/68ID2531105E263/html/SBCC_GAM.html]

 


 **SBCC** and **GAM** 
  
Found 5 abstracts in PubMed, retrieved 05.  
 

 What does Google say? 
 PDF only 
| .edu only 

---

**Interactive relationship** (e.g. stimulation, inhibition, etc)

**Neutral relationship**- Mutations that inactivate  **sbcC**  improve the ability to propagate lambda red  **gam**  phage that carry a palindromic sequence in their DNA.  Ref: 1531222 J Bacteriol, 1992
- Interaction between the  **sbcC**  gene of Escherichia coli and the  **gam**  gene of phage lambda.  Ref: 2531105 Genetics, 1989
- These results indicate that Red or  **Gam**  activities mediate de novo synthesis of PLM in sbcB  **sbcC**  mutants.  Ref: 2148608 Mol Gen Genet, 1990
- The  **sbcC**  gene product of Escherichia coli interferes with the growth of a lambda red  **gam**  phage carrying a long palindrome in its DNA.  Ref: 2530497 Nucleic Acids Res, 1989
- These results suggest that the  **Gam**  protein of lambda, known to interact also with E. coli s recBCD protein, can interact with the product of the  **sbcC**  gene.  Ref: 2531105 Genetics, 1989

**Non-interactive relationship** (e.g. studied together, co-existance, homology, etc.)

- **gam**  mutants of phage lambda carrying long palindromes fail to form plaques on wild type Escherichia coli but do grow on strains that are mutant in the  **sbcC**  gene.  Ref: 2531105 Genetics, 1989
- Examination of plasmid DNA preparations from sbcB  **sbcC**  mutants expressing  **gam**  or red reveals the presence of two molecular species that may represent intermediates in the PLM biosynthesis pathway single branched circles sigma structures and PLM with single stranded DNA tails.  Ref: 2148608 Mol Gen Genet, 1990
- In order to determine whether PLM formation in sbcB  **sbcC**  mutants occurs by conservative break join recombination of circular plasmids or by de novo DNA synthesis, thyA sbcB  **sbcC**  mutants were transferred from thymine to 5 bromo 2 deoxyuridine BUDR supplemented medium, concurrently with induction of red or  **gam**  expression, and the density distribution of plasmid molecular species was analyzed.  Ref: 2148608 Mol Gen Genet, 1990
